# Comprehensive Evaluation of Nutritional, Physicochemical, and Volatile Profiles of Selected Bovine Head Muscles

**DOI:** 10.3390/foods13244098

**Published:** 2024-12-18

**Authors:** Qihan Liu, Anthony Pius Bassey, Ziyu Li, Guanghong Zhou, Xia Fan, Keping Ye

**Affiliations:** 1 State Key Laboratory of Meat Quality Control and Cultured Meat Development, Nanjing Agricultural University, Nanjing 210095, China; 2020108079@stu.njau.edu.cn (Q.L.); bassey_ap44@outlook.com (A.P.B.); liziyu1231@163.com (Z.L.); ghzhou@njau.edu.cn (G.Z.); 2 College of Food Science and Technology, Nanjing Agricultural University, Nanjing 210095, China; fanxia@njau.edu.cn

**Keywords:** bovine head muscle, nutritional profile, physicochemical profile, volatile profile, byproduct utilization

## Abstract

This study analyzed the nutritional composition, physicochemical properties, and volatile profiles of three major bovine head muscles—medial pterygoid, masseter, and buccinator—to reduce byproduct resource waste and increase the utilization rate of bovine head to establish a foundation for its industrial use. Compared to tenderloin, which is popular among consumers, these head muscles were found to be rich in collagen (4.90–13.1 mg/g), low in fat (0.39–1.61%), and abundant in free amino acids (143.93–223.00 mg/100 g). Their compact fiber structures, with minimal gaps between myocytes, resulted in lower cooking and press losses, making them suitable for various production processes. Notably, the medial pterygoid and masseter muscles contained high levels of polyunsaturated fatty acids (PUFAs) and lower saturated fatty acids (SFAs), with a PUFA/SFA ratio exceeding 0.45. The buccinator muscle, while containing more volatile organic compounds associated with undesirable odors and bitter amino acids, was not indicative of spoilage. Overall, this study confirmed that bovine head muscles possess high collagen, low fat, and diverse nutritional qualities, making them suitable as premium raw materials for value-added meat products, and their returns will be most economically equal to the meat derived from cattle.

## 1. Introduction

Beef ranks as the third most consumed meat globally, with a production estimate of 61.377 million tons in 2024 [1,2]. China was a major beef importer in 2021, with a total beef import volume of 2.36 million tons, valued at CNY 12.63 billion [3]. For Chinese consumers, beef has now become the third-most consumed meat, following pork and chicken [4]. It serves as an essential source of high-quality dietary proteins, containing amino acids crucial for human metabolism. However, a significant portion of byproducts generated during slaughter and processing, such as the head, bones, and feet—comprising approximately 22.85% of the total bovine live weight—often goes to waste, with the head contributing a notable proportion [5,6]. These byproducts for most countries are typically used in livestock feed or discarded, contributing to environmental pollution. In many regions of China, people are accustomed to consuming bovine heads and even using them to prepare dishes. Therefore, an effective approach to minimize bovine head waste is to process their edible muscles for industrial use, based on sufficient research on bovine heads.

**Figure 1:**
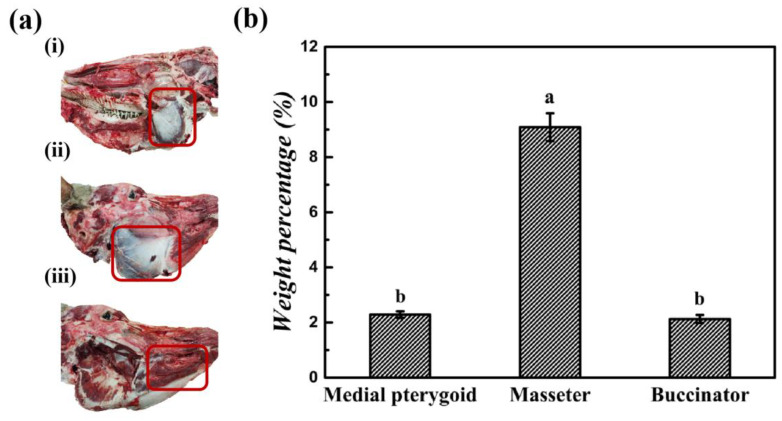
Segmentation and weighing of three parts in bovine head. (**a**) Location (by red box): (**i**) medial pterygoid muscle in the third layer; (**ii**) masseter muscle in first and second layers; (**iii**) buccinator muscle in first layer. (**b**) Weight percentage of different bovine head muscles. a and b on bar indicate significant differences among bovine head muscles (*p* < 0.05), and means and standard error (bars) were plotted (n = 8).

While research on bovine head meat has mainly focused on structural analysis and segmentation, there are several valuable muscles in the head, such as the medial pterygoid, masseter, and buccinator muscles. These muscles have unique structural characteristics, occupying prominent positions within the head [7]. According to Budras et al. [8], the medial pterygoid, located in the third layer and not directly adjacent to the oral cavity, may contribute to its tenderness. The masseter, spread across the first and second layers, is associated with toughness due to its innervation by the deep temporal and masseteric nerves. The buccinator, located in the first layer near the teeth, may have flavor influences from compounds derived from digestion (Figure 1a).

Byproducts such as the tongue and liver were the focus of studies for their potential as high-quality products. For example, Warren et al. [9] highlighted the proteomic and nutritional potential of beef tongue, while Devatkal et al. [10] identified buffalo liver as a rich source of proteins, iron, and zinc. Each byproduct offers distinct nutritional and flavor profiles based on its anatomical location. Despite this, there is limited research on the nutritional and biochemical evaluation of bovine head muscles, which has hindered their development within the beef industry.

This study aims to assess the nutritional composition, physicochemical properties, and volatile profiles of three key bovine head muscles—masseter, medial pterygoid, and buccinator—in comparison to tenderloin, which is preferred by consumers due to its high dietary protein content and tender texture properties [11]. These findings will highlight the nutritional distinctions and provide a qualitative overview of the flavor profiles, making bovine head muscles suitable as premium raw materials instead of expensive beef for diet and value-added meat products, and offer a foundation for promoting the utilization of these valuable byproducts to reduce food waste.

## 2. Materials and Methods

### 2.1. Materials

Eight fresh bovine heads (weighing 53.13 ± 3.87 kg) and corresponding tenderloins (weighing 4.02 ± 0.10 kg), both sourced from male Chinese yellow cattle (393 ± 4.16 kg average) aged 24 months, were procured from the native slaughterhouse (Nanjing, China).

### 2.2. Sample Preparation

Heads and tenderloins were transported to the laboratory in insulated boxes within 1 h. The heads were obtained over three separate batches (two heads, three heads, and three heads) at 3-day intervals. From each head (24 h postmortem), three edible muscles were collected: medial pterygoid muscle (194 ± 3.71 g), masseter muscle (772 ± 15.2 g), and buccinator muscle (181 ± 4.38 g). Tenderloins were used as controls to validate the parameters for bovine head muscles. Each muscle was cut into three uniform pieces (~50 g each) for testing pH, color, moisture content, cooking loss, press loss, texture profile analysis, and Warner–Bratzler shear force on the same day.

The remaining muscle portions were divided into five 6 g samples, vacuum-packed in 7 × 10 cm multilayer coextruded pouches (80 μm thickness, O_2_ permeability of 2.0 cm^3^/m^2^·day·atm at 85% RH/23 °C; Cangzhou Hualiang Packaging and Decoration Co., Ltd., Cangzhou, China) using a DC800-FB-E vacuum chamber (Promax Packaging Solutions Inc., Ontario, USA), and stored at −80 °C until further analysis.

The percentage weight of each muscle was calculated as follows:

(1) $$W(\%)=\frac{W_{1}}{W_{2}}\times 100$$

where *W*_1_ is the weight of each muscle, and *W*_2_ is the weight of bovine head edible muscles.

### 2.3. Nutritional Analysis

#### 2.3.1. Proximate Analysis

Moisture content, crude protein, and crude fat percentages were determined following the official protocols outlined by the Association of Official Analytical Chemists [12]. Collagen content, measured as hydroxyproline concentration, was determined using a hydroxyproline test kit (Beijing Solarbio Science & Technology Co., Ltd., Beijing, China), adhering to the manufacturer’s instructions. The collagen content was calculated using a conversion factor of 7.25 from hydroxyproline, specific to bovine meat cuts [13], and expressed as milligrams of collagen per gram of muscle.

#### 2.3.2. Free Amino Acids

Free amino acids were analyzed following the method described by Oh et al. [14], with slight modifications. In brief, 0.3 g of each meat sample (masseter muscle, medial pterygoid muscle, buccinator muscle, or tenderloin) was homogenized in 1.5 mL of 3% sulfosalicylic acid. The homogenate was centrifuged (12,000× *g*, 15 min, 4 °C) and the supernatant was treated with an equal volume of n-hexane. After phase separation, the solution was filtered through a 0.2 μm filter, and the free amino acid content was determined using a high-speed amino acid analyzer (LA8080 AminoSAAYA, Naka Area Science Laboratory, Hitachi High-Tech Co., Tokyo, Japan) equipped with a BioBasic SCX cation exchange column (4.6 mm × 60.0 mm, 5.0 μm). The results were expressed as milligrams per 100 g of meat (mg/100 g).

#### 2.3.3. Fatty Acids

Fatty acids in the meat samples were analyzed using the method of Tikk et al. [15], with minor modifications. Briefly, 6 g of each sample was homogenized with 20 mL of a chloroform-methanol mixture (2:1, *v*/*v*). The extracted lipids were concentrated using a Pressure Blowing Concentrator (Golden Saqi Technology Co., Ltd., Jinan, China) and subsequently saponified with 2% sodium hydroxide in methanol. The fatty acids were then methylated using 14% boron trifluoride in methanol, dissolved in 7 mL of hexane, and 10 mL of saturated sodium chloride solution was added. After phase separation, the supernatant was filtered using a 0.2 μm filter. The fatty acid methyl esters (FAMEs) were injected into a gas chromatograph (TRACE 1310, Thermo Fisher Scientific, Rodano, Italy) equipped with a TG-FAME column (50 m × 0.25 mm × 0.20 μm). The temperature program began at 80 °C for 1 min, increased to 160 °C at 20 °C/min (held for 1.5 min), then to 205 °C at 3 °C/min (held for 4 min), and finally to 250 °C at 3 °C/min (held for 2 min). Both the injector and detector were set to 280 °C. Fatty acids were identified based on their retention times compared to a FAME standard mix (37 component FAME mix, ANPEL Laboratory Technologies, Shanghai, China), and their concentrations were quantified using the external standard method [16], expressed as mg of FA per 100 g of tissue.

### 2.4. Physicochemical Analysis

#### 2.4.1. pH Measurement

The pH was measured at room temperature using a portable pH meter (Testo 205, Testo AG, Lenzkirch, Germany) equipped with a built-in temperature compensation system. Before each measurement, the device was calibrated with commercial standard buffers (pH 4.0, 7.0, and 10.0; Thermo Fisher Scientific, Singapore). pH readings were taken at three different points of each sample, with three replicate measurements at each point.

#### 2.4.2. Color Measurement

The color of the samples, after blooming for 30 min at 4 °C [17], was measured using a colorimeter (CR-40, Minolta Camera Co., Osaka, Japan) with a 0° viewing angle, 8 mm measurement area, and D65 illuminant. The device was calibrated with a white tile (CR-A43) prior to measurement. Color values for lightness (L*), redness (a*), and yellowness (b*) were recorded from three different surface areas of each sample, with three replicate measurements for each area.

#### 2.4.3. Water Holding Capacity (WHC) Measurement

Cooking loss was determined following the method of Akwetey and Knipe [18], with minor modifications. Muscles were cut into dimensions of 5 cm × 3 cm × 2 cm. Pre-cooking weight was recorded, and after boiling in 72 °C water until the core temperature reached 70 °C, cooking loss (%) was calculated as the percentage of weight loss relative to the initial weight.

Press loss was measured based on the method of Xie et al. [19], with minor modifications. Samples (1 cm × 2 cm × 1 cm) were wrapped in double-layer gauze and 16 layers of filter paper, then placed on a Model YYW-2 Strain Controlled Unconfined Compression Apparatus (Nanjing Soil Instrument Factory Co., Ltd., Nanjing, China). Pre- and post-compression weights were recorded, and press loss (%) was calculated as the percentage of weight loss after compression relative to the initial weight.

#### 2.4.4. Tenderness Measurement

Tenderness was assessed using Texture Profile Analysis (TPA) and Warner–Bratzler shear force (WBSF) tests. The TPA test was performed following the method of Selani et al. [20] with minor modifications, using a Texture Analyzer TA-XT2i (Stable Micro Systems Ltd., Godalming, UK) and the accompanying software (Texture Expert for Windows, Version 1.0). The meat samples (1.5 cm × 1.5 cm × 1 cm) were compressed twice with a 50 mm cylindrical probe (P/50) to 75% of their original height at a constant speed of 1.0 mm/s (pre- and post-test speeds: 5.0 mm/s; trigger force: 5 g; data acquisition rate: 200 pulses per second). The WBSF test was conducted based on the method of Wyrwisz et al. [21], with minor modifications. The meat samples were boiled until the internal temperature reached 70 °C, then cooled to room temperature. Three strips (1.0 cm thick) were cut parallel to the muscle fiber orientation from the core of each sample. Three shear force measurements were taken per strip, resulting in a total of nine measurements per sample. WBSF (N/cm^2^) was measured using a tenderness meter (C-LM3B, Northeast Agricultural University College of Engineering, Harbin, China).

#### 2.4.5. Tissue Structure Measurement

The morphology and structure of the muscle tissue were analyzed using light microscopy, following the method of Maghami et al. [22]. Briefly, meat samples (1 cm × 1 cm × 1 cm) cut perpendicular to the muscle fibers were fixed in 4% paraformaldehyde and soaked in wax to aid tissue fixation. Each sample was then embedded in paraffin using an embedding station (JB-P5, Wuhan Junjie Electronics Co., Wuhan, China) and sectioned with a pathology slicer (RM2016, Leica Instruments Co., Wetzlar, Germany). The sections were stained with hematoxylin and eosin (HE; G1005, Wuhan Servicebio Technology Co., Wuhan, China) and examined under a light microscope (E100, Nikon Co., Tokyo, Japan) at 200× magnification.

### 2.5. Volatile Profile

#### 2.5.1. E-Nose Measurement

The volatile profile of the samples was analyzed using a PEN3 portable E-nose device (Airsense Analytics GmbH, Schwerin, Germany) as described by Bassey et al. [23]. Briefly, each sample (3 g) was minced and placed in a 20 mL vial, then incubated in a water bath at 60 °C for 10 min to intensify odor release before the E-nose probe was inserted into the vial. The analysis parameters were as follows: sampling interval (1.0 s), flush time (200 s), measurement time (60 s), and gas flow rate (400 mL/min). To avoid experimental errors, the probe was cleaned after each analysis. The data were processed using Win-Muster v1.6.2.22 (Airsense Analytics GmbH, Schwerin, Germany), a pattern recognition software.

#### 2.5.2. HS-GC-IMS Measurement

Volatile organic compounds (VOCs) were detected using a GC-IMS instrument (FlavourSpec^®^, G.A.S., Dortmund, Germany) as described by Wang et al. [24]. A 3 g sample was placed in a 20 mL headspace vial (Supelco Inc., Pennsylvania, USA) and sealed. After incubation at 60 °C for 10 min, the sample was transferred into an FS-SE-54-CB capillary column (15 m × 0.53 mm) with high-purity nitrogen (99.99%) as the carrier gas. The GC-IMS analysis conditions were as follows: filling stroke volume (1 mL), injection flow rate (10 mL/min), total analysis time (31 min), injection and post-injection purge times (5 s and 200 s, respectively), pre- and post-injection dwell times (0.5 s each), injection penetration depth (45 mm), sample vial depth (15 mm), and syringe temperature (85 °C). The analyses were carried out at 60 °C, and the resulting compounds were ionized using a 3H ionization source (300 MBq activity, positive ion mode). The ions were driven through a 9.8 cm drift tube under a constant voltage of 5 kV and at 45 °C. The drift gas flow rate (99.9% nitrogen) was 150 mL/min, with 12 scans averaged for each spectrum. The retention index (RI) was calculated using n-ketones (C4–C9, Sinopharm Chemical Reagent, Beijing, China) as an external reference. The VOCs were identified based on drift time and RI by comparing them to the GC-IMS library.

### 2.6. Sensory Evaluation

Sensory evaluation of the muscle samples was conducted by a panel of 8 trained evaluators (4 males and 4 females, 21–24 years old) from Nanjing Agricultural University, selected for their sensory abilities and experience in meat evaluation. Muscle samples were boiled until the internal temperature reached 72 °C, then randomly coded with three-digit numbers and presented to the panelists on disposable white plates. The evaluation was conducted in three batches, with two to three samples evaluated per batch by the same panelists (n = 8). Panelists rated the color, odor, flavor, tenderness, and juiciness of the cooked samples using a 9-point hedonic scale (7–9 = tend to like, 4–6 = neutral, 1–3 = tend to dislike). Water and soda crackers were provided to cleanse the palate between samples [25].

### 2.7. Statistical Analysis

Differences between variables were analyzed using a one-way analysis of variance (ANOVA) with SAS 8.0 (SAS Institute Inc., Cary, NC, USA). The VOCs were qualitatively analyzed using the laboratory analytical viewer (LAV) and the NIST2014 and IMS databases in the GC-IMS Library. GC-IMS data, including principal component analysis and radar plots of E-nose results, were processed using SIMCA software (Version 14.1, Umetrics, Sweden). Variations in the nutritional and physicochemical parameters of different bovine muscles were modeled using linear mixed models, with muscle type as a fixed factor and biological replicates (n = 8) and batch effects as random factors. The results are expressed as mean ± standard error, and statistical significance was set at *p* < 0.05.

## 3. Results and Discussion

### 3.1. Bovine Head Muscle Weights

After segmentation of the bovine heads, the average weight of each head post-debarking and deboning was 8.5 kg. The specific muscles, the medial pterygoid, masseter, and buccinator, accounted for 2.29%, 9.08%, and 2.12% of the total weight, respectively (Figure 1b). Given their higher edible proportion and the more clearly outlined shape among bovine head muscles, we selected the medial pterygoid, masseter, and buccinator muscles as experimental samples for further study.

### 3.2. Nutritional Composition

#### 3.2.1. Proximate Composition Analysis

Table 1 presents the moisture, crude protein, crude fat, and collagen contents of the bovine head muscles compared to tenderloin (control group). The results indicated that the medial pterygoid muscle (76.4%) and masseter muscle (75.9%) had significantly higher moisture contents (*p* < 0.05) than both tenderloin (74.6%) and buccinator muscle (74.1%). No significant differences (*p* > 0.05) in protein content were observed among the groups. Meanwhile, the crude fat content of bovine head muscles was significantly lower than that of tenderloin (*p* < 0.05), particularly in the masseter muscle (0.39%). Cho et al. [26] noted that tenderloin generally had higher protein content than most other beef parts, and the protein content results of bovine head muscles were similar to that of tenderloin, suggesting the excellent quality of bovine head meat. With their high protein and low fat content, bovine head muscles represent a healthy and nutritious option for children, adolescents, and the elderly [27]. Collagen was the most abundant protein (30%) in animals. It made up the connective tissue, providing the framework for muscle and adipose tissue development [28], which was an integral factor influencing beef tenderness. Results showed that the collagen content of the bovine head muscles was significantly higher (*p* < 0.05) than the tenderloin, with buccinator muscle detected with the highest content (13.1%). The tenderest muscles, such as tenderloin and striploin, had low insoluble collagen, whereas the shank meat and neck contained the most insoluble collagen [29]. The results might indicate that the bovine head muscles with higher collagen in a lot of intermolecular cross-linkages were tougher than tenderloin.

#### 3.2.2. Free Amino Acid Composition Analysis

Free amino acids play a crucial role not only in the formation of aromatic compounds but also in assessing the nutritional value of muscle-based foods [30]. Notably, 17 different free amino acids were identified in each group (Table 2). The total amino acid content in the medial pterygoid and masseter muscles was significantly higher than in tenderloin (*p* < 0.05), with alanine, threonine, and glutamic acid being particularly enriched. This finding aligned with previous reports suggesting that these amino acids were predominant in beef [31]. Specifically, alanine and threonine levels in the medial pterygoid and masseter muscles were significantly higher than in the buccinator muscle and tenderloin. Similarly, masseter muscle showed the highest glutamic acid content among the groups, while tenderloin had the lowest (*p* < 0.05). Moreover, essential amino acids such as valine, methionine, and histidine, which humans cannot synthesize, were present at significantly higher levels in the bovine head muscles compared to tenderloin (*p* < 0.05). From a nutritional perspective, these results highlight that bovine head muscles are rich in both essential and non-essential amino acids required by humans.

It is well-established that different amino acids contribute to meat flavor [32]. In this study, the levels of umami and bitter amino acids were significantly higher in the bovine head muscles than in tenderloin, while the buccinator muscle had the lowest content of sweet amino acids (*p* < 0.05). This variation in amino acid profiles likely influenced the development of distinct flavor characteristics, contributing to the unique flavor profile of the bovine head muscles compared to tenderloin.

#### 3.2.3. Fatty Acids Composition Analysis

The fatty acid profile is a critical index for assessing the nutritional value of lipids in food [33]. Table 3 presents the fatty acid composition of the samples. Significant levels (*p* < 0.05) of saturated (1476 ± 10.6), unsaturated (1808 ± 14.0), and monounsaturated (1524 ± 8.49) fatty acids were found in buccinator muscle, while the medial pterygoid muscle showed the highest polyunsaturated fatty acid content (*p* < 0.05). Among the identified fatty acids, oleic acid, stearic acid, and palmitic acid comprised 33.7%, 20.8%, and 19.4% of the total free fatty acids, respectively.

Notably, the concentration of oleic acid accounted for approximately half of the total free fatty acid content in both buccinator muscle and tenderloin, which may contribute to hypocholesterolemic effects by increasing plasma HDL levels [34]. Certain functional unsaturated fatty acids, such as eicosatrienoic acid and arachidonic acid, are beneficial for nutrient metabolism, immune regulation, gene expression modulation, and disease prevention. Additionally, ω-3 fatty acids, including α-linolenic acid and docosahexaenoic acid, were significantly higher in the medial pterygoid and masseter muscles compared to the buccinator muscle and tenderloin (*p* < 0.05). These fatty acids are essential for reducing blood lipid levels and are vital for human health [35].

The PUFA/SFA ratio is an important index for evaluating the nutritional quality of fat for human consumption. According to the World Health Organization, the recommended PUFA/SFA ratio should exceed 0.45 [36]. In this study, the values observed for the medial pterygoid and masseter muscles were 0.81 and 0.85, respectively, indicating high-quality meat for human health. The WHO/FAO also recommends that the ratio of n-6 to n-3 PUFAs in the diet should be between 4 and 6, while in the Chinese diet, this ratio can reach as high as 10, reflecting a significant deficiency of n-3 PUFAs [37]. The n-6 to n-3 PUFA ratios in the masseter and buccinator muscles were 3.05 and 3.42, respectively, which were below the WHO/FAO recommended range, suggesting that bovine head muscles could be a valuable source of n-3 PUFAs for the Chinese diet. Furthermore, the higher oleic acid content in the buccinator muscle suggests enhanced fatty acid biosynthesis in this tissue compared to others [38]. Oleic acid was prone to oxidation, producing various flavor compounds such as ketones, aldehydes, and acids, which might contribute to the distinctive flavor profile of buccinator muscle [38].

### 3.3. Physicochemical Profiles

#### 3.3.1. pH, Color, WHC, and Tenderness

**Figure 2:**
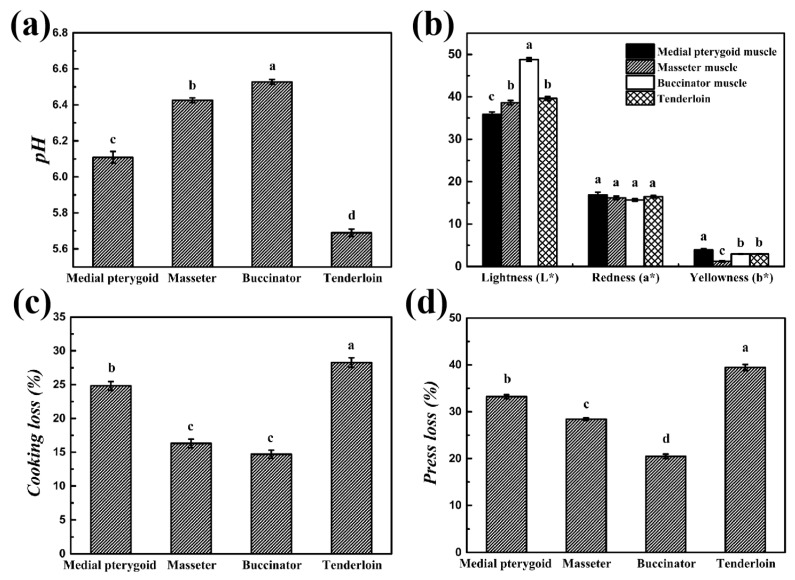
Meat quality characteristics of different parts of bovine meat cuts. (**a**) PH; (**b**) meat color; (**c**) cooking loss; (**d**) press loss. a–d on bar indicates significant differences among groups (*p* < 0.05), and means and standard error (bars) were plotted (n = 8).

The pH of the bovine head muscles was higher than tenderloin (*p* < 0.05) (Figure 2a) and others. Head meat is considered a fast-growing meat compared with the other body parts of cattle. It was previously reported that fast-growing meat demonstrated a higher ultimate pH value, which might be due to lower glycogen content in fast-growing meat [39]. Zhang, Farouk, Young, Wieliczko, and Podmore [40] asserted that high-pH meat exhibited a significant uptrend in WHC and lowered myofibrillar protein solubility than low-pH meat.

Meat color is the first criterion for consumers to judge meat acceptability and quality [41]. Results showed that both the lightness (L*) and yellowness (b*) values were significantly distinct among the groups (*p* < 0.05) (Figure 2b). Buccinator muscle had the highest lightness value (48.8 ± 0.41), due to its whitish appearance, while medial pterygoid muscle demonstrated the lowest (35.9 ± 0.60), which may be linked to a larger diameter in the Type-I muscle fibers and an increase of myoglobin in the muscle tissue [42,43].

Notwithstanding, cooking loss and press loss parameters were affected by meat composition, pH, and WHC [44]. The findings showed that tenderloin had a higher cooking loss and press loss, while the bovine head muscles exhibited a relatively lower loss (Figure 2c,d), resulting in the best WHC (*p* < 0.05). These results might be linked to the abundant collagen content and higher pH values in the bovine head. Purslow [45] reported that meat byproducts contained higher collagen and other connective tissue proteins, and higher pH combined with the latter may induce the increase in the WHC.

Table 4 outlined the result of shear force and the texture profile in the samples. Overall, buccinator muscle exhibited higher hardness, springiness, cohesiveness, and chewiness (*p* < 0.05). These results indicated that the tenderness of the bovine head muscles was relatively low, which should be considered as one of the improvement factors in subsequent processing for its industrialization. Similarly, buccinator muscle and medial pterygoid muscle possessed the highest (121 ± 1.00) and lowest (34.8 ± 0.68) shear force values in WBSF (*p* < 0.05). The differences in meat tenderness and textural properties were caused by the variations in the arrangement structure and diameter of the muscle fibers, illustrating that the muscle structure could be altered by processing techniques to improve the tenderness of the bovine head muscles [46].

#### 3.3.2. Microstructure Analysis

**Figure 3:**
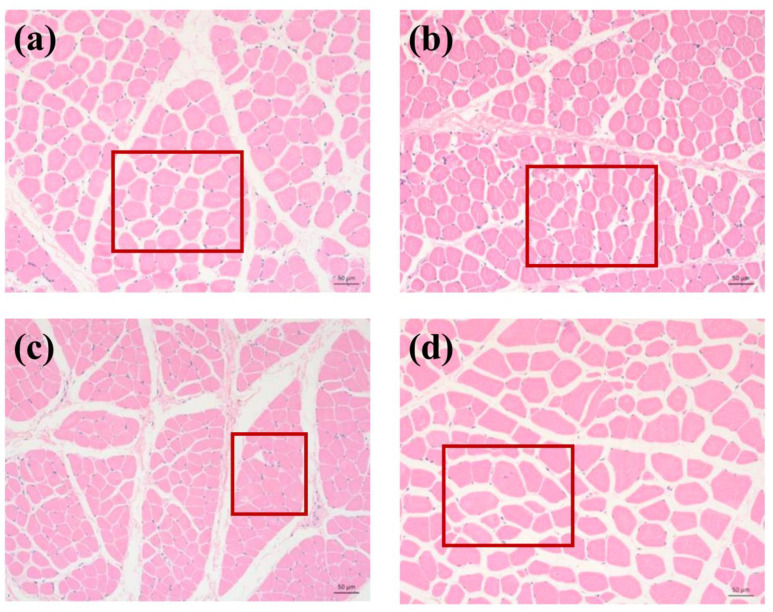
Cross section of muscle fibers in different parts of bovine meat cuts: (**a**) medial pterygoid muscle; (**b**) masseter muscle; (**c**) buccinator muscle; and (**d**) tenderloin.

The microstructure changes in the different bovine meat cuts were determined using light microscopy (Figure 3). The results showed that the bovine head muscles had a more compact fiber structure than tenderloin, and the gaps between adjacent myocytes of masseter muscle and buccinator muscle were narrower than others. Physiological and biochemical reactions in the muscle of different parts would affect the morphology and structure of the muscle tissue to change the water retention of the meat, which explained that the water retention of the bovine head meat was visibly better than that of the tenderloin [47]. As is consistent with the results displayed by meat quality characteristic values, the increased gap in tenderloin and medial pterygoid muscle could improve the cooking loss and press loss and strengthen the tenderness of the meat.

### 3.4. Volatile Profile via E-Nose and HS-GC-IMS

#### 3.4.1. E-Nose Analysis

**Figure 4:**
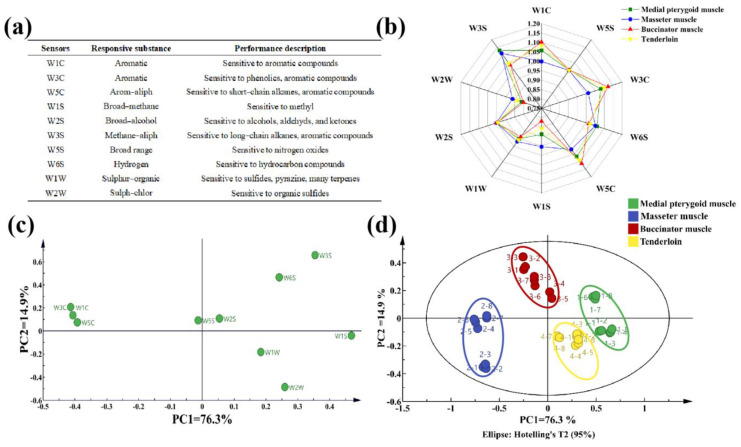
Performance description of electronic nose response data of groups. (**a**) Electronic nose sensors; (**b**) radar chart; (**c**) principal component analysis (PCA) loading plot; (**d**) principal component analysis (PCA) score plot.

The E-nose was sensitive to odors, and their distinctions are ascertained by the response of its sensors. The PEN3 system is equipped with ten sensors, each selectively sensitive to specific volatile organic compounds (VOCs) (Figure 4a,b). Compared to the tenderloin, W1C, W3C, and W5C sensors exhibited a stronger response in the buccinator muscle, indicating the prevalence of aromatic compounds. On the other hand, the medial pterygoid muscle was highly sensitive to W6S and W3S sensors, suggesting the dominance of hydrocarbon, phenolic, and heterocyclic compounds.

The data were further subjected to principal component analysis (PCA) to explain the distinction among the groups and provide insights into the variables that mainly influenced their spatial distributions. The total PC score was 91.2%, indicating a detailed expression of the VOCs in the groups (Figure 4c,d). Considering the spatial distribution in the bovine head muscles, W3S, W3C, and W1C sensors were the most sensitive. The results illustrated that the bovine head muscles were well distinguished in PC1, while the masseter muscle and buccinator muscle were distinct from others, suggesting their significant variation from tenderloin.

#### 3.4.2. HS-GC-IMS Analysis

**Figure 5:**
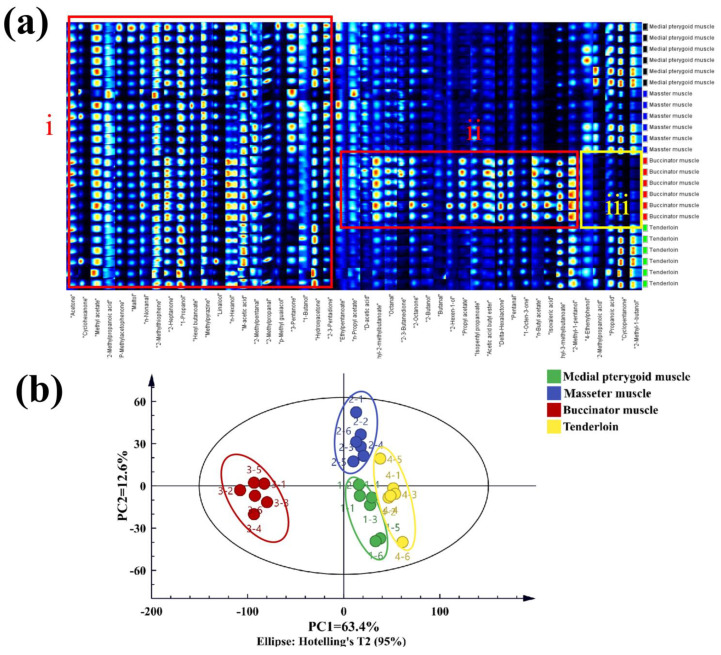
Description of characteristic volatile flavor compounds in different parts of bovine meat cuts. (**a**) HS-GC-IMS fingerprints of compounds; (**b**) principal component analysis (PCA) score plot.

The VOCs were compared to determine their concentrations in the groups (Figure 5a). The “red” highlighted parts signify the common VOCs (i) and distinct VOCs (ii) in all groups, while the “yellow” highlighted part represents the least contents (iii) in the groups. Additionally, the PCA results denote that the closer the groups, the greater the similarities in the VOCs (Figure 5b). Notably, only the buccinator muscle was observed on the negative PC1 axis, illustrating its distinction from other groups hence corroborating the E-nose result.

As shown in Table 5, there were 45 compounds identified among each bovine head muscle, including 11 ketones, 11 esters, 8 alcohols, 6 aldehydes, 4 acids, and 5 other compounds. Generally, the role of ketones, esters, and aldehydes was significant in meat flavor [48]. Ketones were produced from amino acid denaturation and lipid oxidation, which exhibited fruity and creamy flavor attributes [49]. Marked ketones (*p* < 0.05) were detected in the buccinator muscle, including 2-heptanone, 2-octanone, 1-octen-3-one, and 2, 3-butanedione. Nonetheless, p-methylacetophenone, 2, 3-pentadione, and 3-pentanone were abundant in the medial pterygoid muscle, while hydroxyacetone and cyclopentanone dominated in the tenderloin. Huan, Zhou, Zhao, Xu, and Peng [50] asserted that 3-pentanone and 2-hexanone exerted apple-like and floral scents, which took a pleasant odor of tenderloin for consumers. Esters exhibited sweet and fruity aromas from carboxylic acid and alcohol esterification [51]. Of the 11 identified esters in the groups, 8 esters were prevalent in the buccinator muscle, which made the distinctive flavor of the buccinator muscle. Significant aldehydes contents in the buccinator muscle were observed in 2-methylpentanal, nonanal, butanal, pentanal, and octanal (*p* < 0.05). Particularly, common aldehydes in meat products, such as hexanal, octanal, nonanal, and heptanal, were mainly generated from the degradation of unsaturated fatty acids [52]. However, in fresh meat, they were primarily formed from the dehydrogenation of secondary alcohols or lipolysis, mainly engendering pungent caramel, fatty, and butter odor, which might result in unacceptance by consumers [53]. Overall, significant VOCs were detected from the buccinator muscle; this result might be attributed to the contents of oleic acid [38]. The oleic acid oxidized to the flavor compounds, including several “unpleasant” compounds, which might impact consumers’ acceptability more than the other muscles.

### 3.5. Sensory Analysis

As shown in Table 6, different parts of the bovine head muscles had different sensory attributes of color, odor, flavor, tenderness, and juiciness. The odor and flavor characteristics of the bovine head muscle scores were lower than those of tenderloin (*p* < 0.05). The results of these studies were analogous to the report by Miller et al. [54], where cardboardy, sour aromatic, and liver-like flavor attributes were disliked by consumers. Particularly, the buccinator muscle, given its proximity to the mouth, contained higher levels of flavor compounds. And oleic acid oxidized to the flavor compounds, including several “unpleasant” compounds, resulting in the lowest score for its odor and flavor. The tenderloin and medial pterygoid muscle had highly significant scores on tenderness and juiciness, and the masseter muscle and buccinator muscle had significantly low scores on color (*p* < 0.05), which might link to proximate composition and content. Considering all sensory characteristics evaluated, the medial pterygoid muscle and masseter muscle on the bovine head were considered acceptable for customers. Subsequently, for better processing, curing or seasoning can be utilized to improve the tenderness and flavor of bovine head meat thereby preparing high-value byproducts.

## 4. Conclusions

The study confirmed that bovine head muscles contained high collagen and low crude fat content, particularly in medial pterygoid muscle, which was rich in amino acids and polyunsaturated fatty acids. The crude protein content of the bovine head muscles was as much as tenderloin. Additionally, the bovine head muscles had a compact fiber structure to make low cooking loss and press loss, making them suitable for production processes to improve the production rate, and showed an uptrend in texture and shear force. Particularly, the buccinator muscle exhibited significant hardness and shear force. However, the volatile profile revealed different proportions of VOCs among the bovine head muscles and tenderloin, and the buccinator muscle was detected with more distinct VOCs of some “unpleasant” odor, such as nonanal, butanal, and octanal. Overall, the diverse nutritional traits observed in the bovine head muscles indicated the potential for edible value. To make them economically equal to the meat derived from cattle and obtain the favor and trust of customers, the bovine head muscles need to be processed from two aspects of texture and flavor, which should be a potential avenue for bovine head byproduct utilization into the diet or high-value-added products in the beef processing industry.

## Figures and Tables

**Table 1:** Proximate composition of bovine head muscles and tenderloin. Values expressed as means ± standard error (n = 8). Values in same row with different superscripted letters (a–d) signify significant differences (*p* < 0.05).

Composition	Medial Pterygoid Muscle	Masseter Muscle	Buccinator Muscle	Tenderloin
Moisture (%)	76.4 ± 0.22 ^a^	75.9 ± 0.28 ^a^	74.1 ± 0.51 ^b^	74.6 ± 0.31 ^b^
Crude protein (%)	21.9 ± 0.57 ^a^	22.0 ± 0.36 ^a^	22.9 ± 0.53 ^a^	23.1 ± 0.39 ^a^
Crude fat (%)	1.61 ± 0.05 ^b^	0.39 ± 0.14 ^d^	0.73 ± 0.03 ^c^	2.26 ± 0.06 ^a^
Collagen (mg/g)	4.90 ± 0.03 ^c^	8.97 ± 0.14 ^b^	13.1 ± 0.23 ^a^	1.94 ± 0.02 ^d^

**Table 2:** Free amino acid contents of bovine head muscles and tenderloin. Values expressed as means ± standard error (n = 8). Values in same row with different superscripted letters (a–d) signify significant differences (*p* < 0.05).

Amino Acids (mg/100 g)	Medial Pterygoid Muscle	Masseter Muscle	Buccinator Muscle	Tenderloin
Aspartic acid	2.47 ± 0.12 ^a^	1.64 ± 0.27 ^b^	2.07 ± 0.03 ^ab^	0.43 ± 0.00 ^c^
Threonine	38.3 ± 0.19 ^b^	50.9 ± 0.60 ^a^	23.3 ± 0.22 ^d^	34.9 ± 0.30 ^c^
Serine	7.90 ± 0.10 ^a^	6.09 ± 0.13 ^b^	6.75 ± 0.15 ^b^	4.37 ± 0.16 ^c^
Glutamic acid	23.2 ± 0.90 ^b^	30.6 ± 0.40 ^a^	23.4 ± 0.55 ^b^	10.9 ± 0.36 ^c^
Glycine	9.20 ± 0.34 ^ab^	7.88 ± 0.18 ^b^	8.20 ± 0.32 ^b^	10.3 ± 0.09 ^a^
Alanine	71.8 ± 0.89 ^a^	49.5 ± 0.34 ^b^	37.1 ± 0.05 ^c^	37.4 ± 0.30 ^c^
Cystine	0.64 ± 0.01 ^b^	0.49 ± 0.01 ^c^	0.82 ± 0.02 ^a^	0.52 ± 0.01 ^c^
Valine	6.87 ± 0.35 ^a^	6.68 ± 0.17 ^a^	5.41 ± 0.11 ^b^	4.14 ± 0.04 ^c^
Methionine	2.69 ± 0.08 ^a^	1.76 ± 0.05 ^b^	1.48 ± 0.01 ^c^	1.04 ± 0.16 ^d^
Isoleucine	4.87 ± 0.30 ^a^	4.07 ± 0.03 ^a^	2.44 ± 0.07 ^b^	2.42 ± 0.01 ^b^
Leucine	7.93 ± 0.22 ^a^	6.65 ± 0.23 ^b^	4.70 ± 0.10 ^c^	4.12 ± 0.04 ^c^
Tyrosine	4.51 ± 0.10 ^a^	4.03 ± 0.11 ^b^	3.12 ± 0.01 ^c^	3.87 ± 0.01 ^b^
Phenylalanine	7.30 ± 0.10 ^a^	7.44 ± 0.31 ^a^	5.04 ± 0.01 ^b^	5.31 ± 0.04 ^b^
Lysine	12.6 ± 0.74 ^a^	8.85 ± 0.24 ^b^	5.59 ± 0.06 ^c^	4.54 ± 0.01 ^c^
Histidine	8.36 ± 0.14 ^a^	6.62 ± 0.06 ^b^	5.47 ± 0.03 ^c^	4.44 ± 0.04 ^d^
Arginine	10.9 ± 0.60 ^a^	6.46 ± 0.19 ^b^	5.77 ± 0.02 ^bc^	4.39 ± 0.01 ^c^
Proline	3.49 ± 0.13 ^a^	2.86 ± 0.08 ^b^	3.33 ± 0.04 ^a^	2.84 ± 0.04 ^b^
UAA	25.7 ± 1.02 ^b^	32.2 ± 0.67 ^a^	25.4 ± 0.58 ^b^	11.3 ± 0.36 ^c^
SAA	131 ± 1.65 ^a^	117 ± 1.33 ^b^	78.7 ± 0.77 ^d^	89.9 ± 0.89 ^c^
BAA	65.9 ± 2.63 ^a^	52.6 ± 1.39 ^b^	39.0 ± 0.41 ^c^	34.3 ± 0.21 ^d^
EAA	80.5 ± 1.98 ^b^	86.4 ± 1.62 ^a^	48.0 ± 0.57 ^d^	56.5 ± 0.45 ^c^
NEAA	139 ± 3.19 ^a^	113 ± 1.69 ^b^	92.6 ± 1.18 ^c^	76.6 ± 0.98 ^d^
TFAA	223 ± 2.61 ^a^	202 ± 0.22 ^b^	144 ± 0.87 ^c^	136 ± 0.80 ^d^

UAA: umami amino acids (aspartic acid, glutamic acid); SAA: sweet amino acids (glycine, alanine, serine, proline, threonine); BAA: bitter amino acids (lysine, methionine, valine, isoleucine, leucine, tyrosine, histidine, arginine, phenylalanine); EAA: essential amino acids (threonine, valine, methionine, isoleucine, leucine, phenylalanine, lysine); NEAA: non-essential amino acids (aspartic acid, serine, glutamic acid, glycine, alanine, cystine, tyrosine, histidine, arginine); TFAA: total free amino acids.

**Table 3:** Fatty acid contents of bovine head muscles and tenderloin. Values expressed as means ± standard error (n = 8). Values in same row with different superscripted letters (a–d) signify significant differences (*p* < 0.05).

Fatty Acid (mg/100 g Tissue)	Carbon	Medial Pterygoid Muscle	Masseter Muscle	Buccinator Muscle	Tenderloin
Myristic	C14:0	21.6 ± 0.51 ^c^	18.6 ± 0.23 ^c^	65.2 ± 1.33 ^a^	48.1 ± 1.75 ^b^
Pentadecanoic	C15:0	6.84 ± 0.20 ^b^	2.41 ± 0.11 ^c^	7.72 ± 0.32 ^ab^	8.12 ± 0.19 ^a^
Palmitic	C16:0	388 ± 10.5 ^c^	215 ± 7.46 ^d^	802 ± 4.24 ^a^	562 ± 5.30 ^b^
Margaric	C17:0	16.1 ± 0.32 ^d^	20.0 ± 0.41 ^c^	27.1 ± 0.52 ^a^	23.3 ± 0.66 ^b^
Stearic	C18:0	664 ± 14.0 ^a^	309 ± 5.70 ^c^	572 ± 9.90 ^b^	570 ± 2.85 ^b^
Arachidic	C20:0	3.78 ± 0.23 ^a^	1.51 ± 0.02 ^b^	2.48 ± 0.04 ^b^	4.78 ± 0.34 ^a^
	ΣSFA	1100 ± 25.7 ^c^	567 ± 16.4 ^d^	1476 ± 10.6 ^a^	1216 ± 3.39 ^b^
Myristoleic	C14:1 (n−5)	1.94 ± 0.22 ^c^	1.88 ± 0.19 ^c^	11.2 ± 0.32 ^a^	4.73 ± 0.22 ^b^
Cis-10-Pentadecenoic	C15:1 (n−5)	11.1 ± 0.63 ^a^	7.76 ± 0.34 ^b^	2.83 ± 0.22 ^c^	3.85 ± 0.25 ^c^
Palmitoleic	C16:1 (n−7)	18.9 ± 0.18 ^c^	21.1 ± 1.58 ^c^	104 ± 1.30 ^a^	41.6 ± 0.54 ^b^
Oleic	C18:1 (n−9)	526 ± 11.1 ^d^	635 ± 11.4 ^c^	1399 ± 8.80 ^a^	860 ± 6.75 ^b^
Linoleic	C18:2 (n−6)	666 ± 13.5 ^a^	310 ± 14.2 ^c^	194 ± 5.69 ^d^	367 ± 4.95 ^b^
Gondoic	C20:1 (n−9)	2.91 ± 0.07 ^b^	2.46 ± 0.30 ^b^	6.88 ± 0.58 ^a^	5.97 ± 0.20 ^a^
α-Linolenic	C18:3 (n−3) (ALA)	8.59 ± 0.30 ^a^	8.97 ± 0.22 ^a^	4.62 ± 0.33 ^b^	5.41 ± 0.33 ^b^
Cis-11,14-Eicosadienoic	C20:2 (n−6)	3.54 ± 0.07 ^a^	2.40 ± 0.09 ^b^	2.09 ± 0.11 ^b^	1.48 ± 0.06 ^c^
Eicosatrienoic	C20:3 (n−6)	19.0 ± 0.36 ^a^	16.4 ± 0.76 ^b^	8.13 ± 0.33 ^d^	12.52 ± 0.08 ^c^
Arachidonic	C20:4 (n−6) (ARA)	193 ± 2.95 ^a^	139 ± 4.74 ^b^	73.6 ± 1.00 ^c^	85.9 ± 3.85 ^c^
Docosahexaenoic	C22:6 (n−3) (DHA)	1.67 ± 0.01 ^a^	1.29 ± 0.06 ^b^	1.07 ± 0.05 ^b^	1.19 ± 0.05 ^b^
	ΣUFA	1453 ± 29.4 ^b^	1147 ± 14.7 ^c^	1808 ± 14.0 ^a^	1389 ± 12.2 ^b^
	ΣMUFA	561 ± 12.2 ^d^	668 ± 12.8 ^c^	1524 ± 8.49 ^a^	916 ± 6.43 ^b^
	ΣPUFA	892 ± 17.2 ^a^	479 ± 12.1 ^b^	284 ± 6.29 ^c^	474 ± 6.12 ^b^
	ΣFA	2553 ± 37.5 ^b^	1714 ± 24.7 ^c^	3284 ± 24.6 ^a^	2606 ± 15.6 ^b^
	UFA:FA	0.57 ± 0.01 ^b^	0.67 ± 0.02 ^a^	0.55 ± 0.01 ^b^	0.53 ± 0.01 ^b^
	PUFA:SFA	0.81 ± 0.05 ^a^	0.85 ± 0.04 ^a^	0.19 ± 0.01 ^c^	0.39 ± 0.01 ^b^
	n−6:n−3	4.28 ± 0.12 ^b^	3.05 ± 0.14 ^c^	3.42 ± 0.06 ^c^	5.00 ± 0.18 ^a^

ΣSFA: sum of saturated fatty acids; ΣUFA: sum of unsaturated fatty acids; ΣMUFA: sum of monounsaturated fatty acids; ΣPUFA: sum of polyunsaturated fatty acids; ΣFA: sum of free fatty acids.

**Table 4:** Shear force value and texture profile characteristics of bovine head muscles and tenderloin. Values expressed as means ± standard error (n = 8). Values in same row with different superscripted letters (a–d) signify significant differences (*p* < 0.05).

Parameter	Medial Pterygoid Muscle	Masseter Muscle	Buccinator Muscle	Tenderloin
Hardness (g)	8284 ± 124 ^c^	14,672 ± 59.8 ^b^	34,744 ± 259 ^a^	6749 ± 119 ^d^
Springiness	0.37 ± 0.00 ^c^	0.45 ± 0.01 ^b^	0.52 ± 0.00 ^a^	0.29 ± 0.00 ^d^
Cohesiveness	0.39 ± 0.00 ^c^	0.42 ± 0.00 ^b^	0.67 ± 0.00 ^a^	0.27 ± 0.00 ^d^
Chewiness (g)	1099 ± 19.1 ^c^	2318 ± 37.5 ^b^	10,419 ± 106 ^a^	453 ± 6.40 ^d^
Shear force (N/cm^2^)	34.8 ± 0.68 ^d^	67.7 ± 1.39 ^b^	121 ± 1.00 ^a^	47.1 ± 0.50 ^c^

**Table 5:** Volatile profile of bovine head muscles and tenderloin identified by HS-GC-IMS. Values expressed as means ± standard error (n = 8). Values in same row with different superscripted letters (a–d) signify significant differences (*p* < 0.05).

Kind	Compound	CAS#	Formula	Intensity (V)			
				Medial Pterygoid	Masseter	Buccinator	Tenderloin
Ketones							
	Acetone	67-64-1	C_3_H_6_O	278 ± 24.1 ^a^	356 ± 27.8 ^a^	379 ± 34.0 ^a^	348 ± 33.4 ^a^
	Cyclohexanone	108-94-1	C_6_H_10_O	389 ± 37.2 ^b^	671 ± 49.8 ^a^	375 ± 31.6 ^b^	633 ± 81.2 ^ab^
	p-Methylacetophenone	122-00-9	C_9_H_10_O	750 ± 74.7 ^a^	540 ± 37.7 ^ab^	496 ± 12.7 ^b^	476 ± 13.8 ^b^
	2-Heptanone	110-43-0	C_7_H_14_O	663 ± 58.4 ^a^	391 ± 22.8 ^b^	711 ± 18.6 ^a^	626 ± 39.6 ^a^
	Hydroxyacetone	116-09-6	C_3_H_6_O_2_	2452 ± 182 ^b^	2054 ± 74.7 ^b^	2142 ± 129 ^b^	3279 ± 121 ^a^
	2,3-Pentadione	600-14-6	C_5_H_8_O_2_	1006 ± 42.9 ^a^	945 ± 46.1 ^a^	719 ± 14.6 ^b^	243 ± 26.0 ^c^
	2-Octanone	111-13-7	C_8_H_16_O	198 ± 21.7 ^b^	160 ± 13.8 ^b^	398 ± 20.3 ^a^	187 ± 12.3 ^b^
	1-Octen-3-one	4312-99-6	C_8_H_14_O	55.7 ± 0.62 ^c^	94.0 ± 4.69 ^bc^	298 ± 32.3 ^a^	188 ± 18.7 ^b^
	2,3-Butanedione	431-03-8	C_4_H_6_O_2_	465 ± 24.7 ^b^	567 ± 35.7 ^b^	839 ± 56.8 ^a^	117 ± 13.2 ^c^
	3-Pentanone	96-22-0	C_5_H_10_O	529 ± 11.8 ^a^	504 ± 15.6 ^a^	258 ± 25.9 ^b^	171 ± 21.9 ^b^
	Cyclopentanone	120-92-3	C_5_H_8_O	1788 ± 58.0 ^b^	951 ± 51.8 ^c^	469 ± 35.0 ^d^	2092 ± 53.5 ^a^
Esters							
	Methyl acetate	79-20-9	C_3_H_6_O_2_	895 ± 5.10 ^ab^	892 ± 39.6 ^ab^	939 ± 17.2 ^a^	736 ± 59.2 ^b^
	Hexyl butanoate	263-96-36	C_10_H_20_O_2_	310 ± 26.7 ^a^	333 ± 29.2 ^a^	295 ± 2.73 ^a^	184 ± 6.37 ^b^
	n-Propyl acetate	10-96-04	C_5_H_10_O_2_	80.3 ± 7.31 ^a^	94.0 ± 4.82 ^a^	97.3 ± 4.37 ^a^	42.7 ± 2.72 ^b^
	Ethyl pentanoate	539-82-2	C_7_H_14_O_2_	94.7 ± 5.96 ^a^	93.7 ± 6.12 ^a^	57.7 ± 2.83 ^b^	32.3 ± 2.78 ^c^
	n-Butyl acetate	123-86-4	C_6_H_12_O_2_	67.7 ± 3.47 ^bc^	81.0 ± 4.29 ^b^	299 ± 7.51 ^a^	50.3 ± 0.85 ^c^
	Acetic acid butyl ester	123-86-4	C_6_H_12_O_2_	127 ± 4.49 ^b^	137 ± 4.25 ^b^	454 ± 20.9 ^a^	110 ± 0.85 ^b^
	Propyl acetate	109-60-4	C_5_H_10_O_2_	102 ± 1.87 ^b^	105 ± 1.08 ^b^	643 ± 18.8 ^a^	159 ± 10.5 ^b^
	Delta-Hexalactone	823-22-3	C_6_H_10_O_2_	151 ± 16.0 ^c^	160 ± 12.2 ^c^	1093 ± 44.5 ^a^	490 ± 25.6 ^b^
	Isopentyl propanoate	105-68-0	C_8_H_16_O_2_	17.0 ± 0.41 ^c^	31.3 ± 3.79 ^bc^	69.0 ± 3.21 ^a^	43.0 ± 2.48 ^b^
	Methyl-2-methylbutanoate	868-57-5	C_6_H_12_O_2_	215 ± 24.2 ^b^	139 ± 1.89 ^c^	471 ± 13.6 ^a^	172 ± 9.51 ^bc^
	Ethyl-3-methylbutanoate	108-64-5	C_7_H_14_O_2_	254 ± 26.1 ^c^	193 ± 17.3 ^c^	766 ± 7.06 ^a^	611 ± 25.5 ^b^
Alcohols							
	1-Propanol	71-23-8	C_3_H_8_O	3457 ± 19.5 ^b^	3334 ± 73.9 ^b^	2830 ± 118 ^c^	3951 ± 23.4 ^a^
	Linalool	78-70-6	C_10_H_18_O	994 ± 71.4 ^a^	1210 ± 130 ^a^	948 ± 45.7 ^a^	1227 ± 144 ^a^
	n-Hexanol	111-27-3	C_6_H_14_O	76.3 ± 6.12 ^b^	55.7 ± 3.09 ^b^	118 ± 4.45 ^a^	82.0 ± 6.98 ^b^
	2-Butanol	78-92-2	C_4_H_10_O	77.0 ± 8.16 ^ab^	46.7 ± 4.74 ^b^	96.3 ± 7.14 ^a^	45.0 ± 4.25 ^b^
	2-Hexen-1-ol	2305-21-7	C_6_H_12_O	48.0 ± 4.25 ^b^	38.7 ± 3.32 ^b^	261 ± 26.2 ^a^	61.0 ± 3.56 ^b^
	2-Methyl-1-pentanol	105-30-6	C_6_H_14_O	46.3 ± 3.47 ^c^	40.7 ± 3.68 ^c^	140 ± 4.69 ^a^	105 ± 5.88 ^b^
	1-Butanol	71-36-3	C_4_H_10_O	163 ± 4.57 ^b^	189 ± 21.2 ^b^	59.7 ± 2.87 ^c^	267 ± 19.9 ^a^
	2-Methyl-1-butanol	137-32-6	C_5_H_12_O	324 ± 15.1 ^ab^	302 ± 34.7 ^b^	67.7 ± 3.69 ^c^	423 ± 27.8 ^a^
Aldehydes							
	Nonanal	124-19-6	C_9_H_18_O	175 ± 18.5 ^ab^	151 ± 21.4 ^ab^	195 ± 7.59 ^a^	111 ± 5.55 ^b^
	2-Methylpentanal	123-15-9	C_6_H_12_O	92.7 ± 6.65 ^a^	102 ± 13.8 ^a^	110 ± 11.0 ^a^	104 ± 5.35 ^a^
	Butanal	123-72-8	C_4_H_8_O	118 ± 9.27 ^bc^	187 ± 7.43 ^b^	343 ± 22.7 ^a^	99.0 ± 5.10 ^c^
	Pentanal	110-62-3	C_5_H_10_O	94.7 ± 3.92 ^b^	91.7 ± 3.52 ^b^	428 ± 17.2 ^a^	104 ± 9.59 ^b^
	Octanal	124-13-0	C_8_H_16_O	29.3 ± 2.36 ^b^	32.7 ± 2.72 ^b^	72.0 ± 0.88 ^a^	25.7 ± 2.49 ^b^
	2-Methylpropanal	78-84-2	C_4_H_8_O	319 ± 26.3 ^ab^	414 ± 53.1 ^a^	171 ± 10.2 ^b^	483 ± 64.5 ^a^
Acids							
	2-methylpropanoic acid	79-31-2	C_4_H_8_O_2_	285 ± 14.9 ^a^	86.0 ± 2.04 ^b^	107 ± 13.7 ^b^	250 ± 13.5 ^a^
	acetic acid	64-19-7	C_2_H_4_O_2_	734 ± 12.4 ^ab^	680 ± 28.8 ^b^	721 ± 16.1 ^b^	820 ± 5.27 ^a^
	Isovaleric acid	503-74-2	C_5_H_10_O_2_	35.3 ± 2.95 ^c^	31.0 ± 1.87 ^c^	648 ± 39.3 ^a^	254 ± 19.9 ^b^
	Propanoic acid	79-09-4	C_3_H_6_O_2_	417 ± 28.3 ^a^	360 ± 22.3 ^a^	204 ± 18.2 ^b^	414 ± 20.6 ^a^
Others							
	Maltol	118-71-8	C_6_H_6_O_3_	515 ± 37.4 ^a^	322 ± 27.1 ^b^	296 ± 11.3 ^b^	306 ± 13.0 ^b^
	2-Methylthiophene	554-14-3	C_5_H_6_S	901 ± 5.18 ^a^	866 ± 13.8 ^a^	732 ± 34.2 ^b^	885 ± 10.2 ^a^
	Methylpyrazine	109-08-0	C_5_H_6_N_2_	106 ± 6.08 ^a^	131 ± 7.59 ^a^	129 ± 3.00 ^a^	108 ± 3.94 ^a^
	p-Methyl guaiacol	93-51-6	C_8_H_10_O_2_	289 ± 38.0 ^a^	167 ± 12.0 ^b^	153 ± 4.33 ^b^	144 ± 2.72 ^b^
	Ethenylphenol	2628-17-3	C_8_H_8_O	163 ± 13.5 ^a^	104 ± 12.3 ^b^	95.3 ± 3.98 ^b^	113 ± 8.82 ^ab^

**Table 6:** Sensory data (mean values ± standard errors) of bovine head muscles and tenderloin. Values in same row with different superscripted letters (a–d) signify significant differences (*p* < 0.05).

Composition	Medial Pterygoid Muscle	Masseter Muscle	Buccinator Muscle	Tenderloin
Color	7.13 ± 0.83 ^b^	7.75 ± 1.04 ^a^	4.38 ± 1.19 ^c^	8.13 ± 0.64 ^a^
Odor	7.00 ± 0.76 ^b^	7.25 ± 0.89 ^b^	5.13 ± 1.13 ^c^	7.88 ± 0.64 ^a^
Flavor	7.63 ± 0.74 ^b^	7.13 ± 0.83 ^c^	5.75 ± 1.04 ^d^	8.50 ± 0.53 ^a^
Tenderness	8.38 ± 0.74 ^a^	6.50 ± 0.93 ^b^	5.13 ± 1.36 ^c^	8.13 ± 0.64 ^a^
Juiciness	7.75 ± 0.71 ^a^	6.63 ± 0.52 ^b^	4.88 ± 0.83 ^c^	7.38 ± 0.92 ^a^

## Data Availability

The original contributions presented in this study are included in the article. Further inquiries can be directed to the corresponding author.
